# Neural networks to estimate multiple sclerosis disability and predict progression using routinely collected healthcare data

**DOI:** 10.1177/13524585251347513

**Published:** 2025-07-03

**Authors:** Giuseppina Affinito, Marcello Moccia, Roberta Lanzillo, Ruth Ann Marrie, Jeremy Chataway, Vincenzo Brescia Morra, Raffaele Palladino

**Affiliations:** Department of Public Health, University of Naples Federico II, Naples, Italy; Neurology Unit, Policlinico Federico II University Hospital, Naples, Italy; Department of Molecular Medicine and Medical Biotechnology, University of Naples Federico II, Naples, Italy; Neurology Unit, Policlinico Federico II University Hospital, Naples, Italy; Department of Neuroscience, Reproductive Science and Odontostomatology, University of Naples Federico II, Naples, Italy; Faculty of Medicine, Dalhousie University, Halifax, NS, Canada; Queen Square Multiple Sclerosis Centre, Department of Neuroinflammation, UCL Queen Square Institute of Neurology, Faculty of Brain Sciences, University College London, London, UK; Medical Research Council Clinical Trials Unit at UCL, Institute of Clinical Trials and Methodology, University College London, London, UK; Biomedical Research Centre, National Institute for Health Research, University College London Hospitals, London, UK; Department of Neuroscience, Reproductive Science and Odontostomatology, University of Naples Federico II, Naples, Italy; Multiple Sclerosis Unit, Policlinico Federico II University Hospital, Naples, Italy; Department of Public Health, University of Naples Federico II, Naples, Italy; Department of Primary Care and Public Health, Imperial College, London, UK

**Keywords:** Multiple sclerosis, disability estimation, neural network, EDSS, survival analysis

## Abstract

**Background and Objectives:**

Multiple sclerosis (MS)-related disability is conventionally measured using the Expanded Disability Status Scale (EDSS), which requires neurological examination and is generally embedded in clinical records, making it unavailable in administrative datasets. This limits its utility for population-level estimates and healthcare planning. This study aims to use routinely collected healthcare data to fill this gap.

**Methods:**

We conducted a population-based study using administrative data from Campania Region (Italy) to develop and validate neural network algorithms to estimate MS-related disability and predict its progression (2015–2021). We employed a deep learning approach to estimate the EDSS, and a hybrid model combining survival analysis with neural network predictions to forecast the risk of EDSS progression.

**Results:**

The model estimated EDSS with 0.68 accuracy, 0.68 precision, and 0.67 F1-score. The hybrid model had a predictive performance of 0.92. From 2016 to 2021, 9.01% of the population had EDSS ⩽ 3.0, 62.10% had EDSS between 3.5 and 5.5, and 28.89% had EDSS ⩾ 6.0. Looking at projections from 2021 to 2026, 67.68% people with EDSS ⩽ 3.0 are expected to progress to EDSS 3.5–5.5.

**Conclusion:**

These findings highlight the potential of advanced data analytics using administrative data to improve MS monitoring, healthcare planning, and decision-making.

## Introduction

Multiple sclerosis (MS) is a chronic neuroinflammatory disease, primarily affecting young adults and causing progressive disability.^1–3^ MS disability is typically assessed using the Expanded Disability Status Scale (EDSS), which requires neurological examination and is generally embedded in clinical records, without possibility to derive in administrative databases. This limits its use for population-level estimates and healthcare planning, particularly for patients with higher levels of disability who may have limited access to regular clinical follow-up. Therefore, routinely collected healthcare data may offer an alternative to enable broader monitoring even in the absence of direct clinical assessments.^4^

The EDSS’s non-linearity complicates estimation, causing conflicting results in administrative data studies. Some studies have focused uniquely on severe disability, while others have used analytical methods that do not sufficiently address the non-linear characteristics of the EDSS.^5–7^ Few studies validated algorithms with clinical data, essential for accuracy, and reproducibility.^5,7^

High-granularity data and deep learning can model complex non-linear relationships with high robustness and predictive power.^8^ Deep learning models have been instrumental in forecasting disease outbreaks and guiding interventions by analysing comprehensive datasets, such as epidemiological records, environmental factors, and population demographics.^9,10^ These developments highlight the role of advanced data analytics in medical decision-making. Therefore, using routinely collected healthcare data (2015–2021) from the Campania Region, this study aimed to (1) validate a neural network algorithm to classify EDSS levels; (2) validate a hybrid model predicting EDSS progression; and (3) estimate disability distribution and forecast progression over five years.

## Methods

### Study design

This is a population-based study, obtained from the retrospective analysis of routinely collected. healthcare data of individuals residing in the Campania Region, the largest region of South Italy, from 2015 to 2021 (http://dati.istat.it/).

The Campania Region operates under the universal, publicly funded Servizio Sanitario Nazionale (SSN), ensuring fair access to healthcare and capturing nearly all diagnosed cases.

### Study population

A validated case-finding algorithm identified PwMS in Campania from 2015 to 2021.^11–13^ It is based on the identification of at least one MS record, in any of the following routinely collected healthcare databases:

1. Regional Drug Prescription database, which included all MS-specific prescriptions for disease-modifying treatments (DMTs) during the study period (e.g. alemtuzumab; cladribine tablets; dimethyl fumarate; fingolimod; glatiramer acetate; interferon beta-1a; peg-interferon beta-1a; interferon beta-1b; natalizumab; ocrelizumab; and teriflunomide).
2. Hospital Discharge Record database, which included all admissions in the study period with an International Classification of Disease (ICD-9 CM), with code of MS as one of the discharges diagnoses (340).
3. Outpatient database, which includes all MS-related and non-related outpatient encounters.

Hospital admissions, DMT prescriptions, and outpatient visits delivered outside of the Campania Region were reported to the Campania Region Healthcare Regulatory Society (So.Re.Sa.) for refund purposes and then included in the aforementioned datasets. From the database, individuals with a diagnosis of MS not resident in the Campania Region were excluded. Patient unique identifier codes were fully anonymised by the Campania Region Healthcare Regulatory Society (So.Re.Sa.) before releasing the datasets. As the same anonymisation algorithm was used across datasets, deterministic data linkage was possible at the individual level.

### Clinical dataset

About 30% of the MS population residing in Campania Region is currently registered within the *Multiple Sclerosis Clinical Care and Research Centre* of the University ‘Federico II’ of Naples (Italy).^11^

This population reflects the characteristics of the entire MS population in the Campania region, making it a suitable for validation (Table 1). Therefore, for hospital discharge records, outpatients, and prescription data of the MS population followed-up at this MS Unit, linkage with clinical data was available. We performed the anonymization using the same algorithm adopted for the administrative dataset. Hence, we used the recorded EDSS scores from EDSS-certified neurologists in the clinical dataset to validate the algorithms by comparing the predicted EDSS classifications against clinical scores.^11^

**Table 1.** Table shows comparison of demographic and clinical characteristics between administrative dataset only (*n* = 6,295) and combination of administrative and clinical dataset (*n* = 1,783) from 2015 to 2021.

	Administrative dataset (*n* = 6295)	Clinical dataset (*n* = 1783)	Test statistic *p* value
Age, years (±SD)	42.51 ± 13.85	42.51 ± 11.87	t=0.45 p=0.58
Sex, female (%)	4012 (63.83%)	1154 (64.72%)	$\chi^{2}=0.928$ p=0.335
DMTs (#ITPs/Total #ITPs)			
MABs	1141 (16.07%)	702 (24.04%)	$\chi^{2}=92.31$ p<0.01
Oral	3253 (45.82%)	1172 (40.14%)	
Injectables	2706 (38.11%)	1046 (35.82%)	
Platform	4744 (66.83%)	1636 (56.03%)	$\chi^{2}=108.2$ p<0.01
High-Efficacy	2355 (33.17%)	1284 (43.97%)	
DMTs switches/Total #Platform DMT (Platform to High-Efficacy)	730 (15.39%)	496 (30.32%)	$\chi^{2}=286.95$ p<0.01
Regular hospital admissions (#admissions/ Total #admissions)			
Emergency admissions	1235 (51.12%)	252 (56.15%)	$\chi^{2}=2.103$ p=0.147
MS-related admissions	1204 (49.21%)	195 (44.25%)	$\chi^{2}=31.53$ p<0.01
Outpatient services (#outpatients/Total #outpatients)			
Consultation	11,214 (8.48%)	1547 (9.21%)	$\chi^{2}=27.27$ p<0.01
Diagnostic	13,028 (9.85%)	1507 (8.97%)	
Laboratory	106,874 (80.79%)	13,613 (81.02%)	
Procedure	1137 (0.86%)	131 (0.78%)	
Therapeutic	39 (0.03%)	3 (0.02%)	
Rehabilitation outpatient services (#Rehabilitation outpatients/Total #outpatients)	2455 (2.21%)	611 (4.26%)	$\chi^{2}=234.88$ p<0.01

Data are presented as mean ± standard deviation (SD) for age, as count (%) and proportions for sex and various healthcare parameters. Statistical significance was assessed using *t*-test for age, and chi-square test for categorical variables, with *p* values reported.

DMTs: disease-modifying therapies; MABs: monoclonal antibodies; ITP: individual treatment period.

### Study outcomes

The EDSS is a 10-point scale used to measure disability in individuals with MS. EDSS scores are derived from the combination of impairments in eight functional systems (visual, brainstem, pyramidal, cerebellar, sensory, bowel and bladder, cerebral, and ambulatory). In this study, we identified three EDSS classes: EDSS ⩽ 3; EDSS 3.5–5.5; EDSS ⩾ 6.^14^ An EDSS score of ⩽3 defines patients experiencing no, minimal, or mild disability. Scores between 3.5 and 5.5 indicate moderate to severe disability with limited impairment of activities, while patients with EDSS scores ⩾6 points experience severe disability and require ambulatory aid. This classification is consistent with previous studies and provides a framework to assess transitions across meaningful disability milestones, as it include the progression through EDSS 4.0 and 6.0, which are clinically relevant thresholds of disability.^15,16^

We included relapse-free EDSS values from patients with annual evaluations to ensure data reliability. We standardized the observation period to a 6-month interval because clinical assessments in our dataset are scheduled at least every 6 months, in line with routine MS monitoring practices. This interval starts from the entry date (i.e. the patient’s first recorded interaction in the administrative databases) to ensure consistent data collection while minimizing the impact of irregular follow-ups.

### Study covariates

Study covariates were age, sex, use of DMTs, duration of therapy, number of regular hospital admissions, length of hospital admission, and outpatient services.

DMTs were classified into monoclonal antibodies (MABs: alemtuzumab, natalizumab, ofatumumab, ocrelizumab), tablets (cladribine, dimethyl fumarate, teriflunomide, fingolimod), and injectables (glatiramer acetate, interferon, pegylated-interferon). An additional classification was based on European and Italian Regulatory Agencies using efficacy level (Platform: dimethyl fumarate, glatiramer acetate, interferon, teriflunomide; High-Efficacy: alemtuzumab, cladribine, fingolimod, natalizumab, and ocrelizumab), from which we derived DMTs switches (Platform to High-Efficacy). Hospital admissions were categorized into MS-related/non-MS-related (ICD 9 CM 340), and emergency/non-emergency admissions.^17^ Subsequently, outpatient services were classified into consultation, diagnostic, laboratory, procedure, therapeutic, and rehabilitation by a group of expert neurologists (Supplemental Appendix 1). Finally, specific ICD-9 codes were used to identify the presence of severe disability conditions (Supplemental Appendix 1).

### Statistical analysis

Variables were described as mean (SD) for continuous data or proportions for categorical data. The chi-square test and *t*-test were employed to evaluate the statistical significance of differences in variables between administrative dataset and clinical dataset from 2015 to 2021.

We performed statistical analyses using Stata 18.0 and Python programming language.

#### Estimation of EDSS class

We employed a deep learning approach to estimate the EDSS classes for PwMS using routinely collected healthcare data quantified over a 6-month period. Each participant’s data were collected for 6 months from their respective entry date (first record in the administrative databases). Patients were included only if they had corresponding EDSS information in the clinical dataset, ensuring reliable model validation.

We validated the algorithm using a clinical dataset, which provided EDSS labels for training and testing.

The model used a three-layer feed-forward neural network with rectified linear unit (ReLU) activation functions. The dataset was randomly split into training (70%) and validation (30%). The architecture included a first hidden layer with 64 neurons, a second with 32 neurons, and an output layer with 3 neurons using a SoftMax activation function, for multi-class classification.

Model optimization was performed using the Adam optimizer, with a learning rate of 0.001 and a batch size of 32. Dropout (rate = 0.2) was applied to prevent overfitting, and L2 regularization was incorporated to enhance generalization. Training was conducted over 10 epochs, and hyperparameter tuning was carried out through grid search, testing combinations of learning rates, batch sizes, and the number of neurons in each layer.

We assessed accuracy, precision, recall, and F1-score, along with sensitivity and specificity for each EDSS class.

#### Prediction of risk of disability progression

Following prior approaches, we developed a hybrid model integrating neural networks and survival analysis to predict disability progression.^18,19^

The model input data included patient demographics, such as age and sex and healthcare utilization (hospital admissions, outpatient visits and DMT use). The event was a transition between EDSS classes.

We used the classification algorithm to identify patients with EDSS ⩾ 6.0.

The neural network included three fully connected layers with 32, 32, and 16 neurons, using ReLU activation. Cox proportional hazards model was taken as the loss function while training, enabling the neural network to forecast the event of interest measured in terms of the time to disability progression.^18^

The Adam optimizer iteratively adjusted model parameters over multiple epochs, with early stopping applied when validation loss plateaued to prevent overfitting. We employed five-fold cross-validation splitting into training (60%), validation (20%), and test subsets (20%) to ensure unbiased evaluation.^20–22^ We assessed the predictive accuracy of the hybrid model using the Concordance Index (C-index) and Integrated Brier Score (IBS).

#### Distribution and prediction of disability classes

We applied the first algorithm to the entire cohort to estimate the temporal distribution of disability across the entire population with MS in the Campania Region. In addition, we employed the second algorithm to predict the risk of class change from 2021 to 2026.

k-means clustering was applied to define the thresholds of risk associated with class change. The unsupervised cluster algorithm divided the patients into three categories of risk: low, moderate, and high, based on their predicted cumulative hazard at 5 years.

A Sankey graph visualized disability class transitions from 2021 to 2026.

For all the models developed in this study, patients included in the training phase were not excluded from the subsequent deep learning analyses conducted on the entire MS population. Each model was developed and internally validated using distinct training and testing subsets to ensure robustness and prevent overfitting. After validation, the models were applied to the full dataset to estimate EDSS classes for all eligible patients, ensuring that predictions were consistent and representative of the entire MS population.

#### Standard protocol approvals, registrations, and patient consents

The study was approved by the University ‘Federico II’ of Naples Ethics Committee (332/21). All patients signed informed consent authorizing the use of anonymised, routinely collected healthcare data, in line with data protection regulation (GDPR EU2016/679). The study was performed in accordance with good clinical practice and the Declaration of Helsinki.

## Results

Out of 8642 prevalent PwMS in the Campania Region from 2015 to 2021, we included 8078 PwMS (age 42.63 ± 13.47; females 64%). We excluded 564 PwMS due to missing data in relation to demographics variables. A comparative analysis of key characteristics between the excluded sub-cohort, and the main cohort did not indicate significant differences (Supplemental Appendix 1).

Linkage to the clinical dataset was available for 22.07% of the regional MS population. Demographic and healthcare resource utilization are reported in Table 1.

### Estimation of EDSS class

The accuracy of the algorithm was 0.68 (95% confidence interval [CI]: 0.63, 0.72), indicating that the model correctly predicted the class labels for approximately 68% of the instances. The precision, calculated at 0.68 (95% CI: 0.63, 0.74), indicated the proportion of correctly predicted positive instances out of all instances predicted as positive. Similarly, the recall, with a value of 0.69 (95% CI: 0.64, 0.75), represented the proportion of correctly predicted positive instances out of all actual positive instances. Sensitivity and specificity for each class are shown in Supplemental Appendix 1. Finally, the F1-score which considers both precision and recall, was calculated to be 0.67 (95% CI: 0.61, 0.72).

### Prediction of risk of disability progression

**Figure 1.**
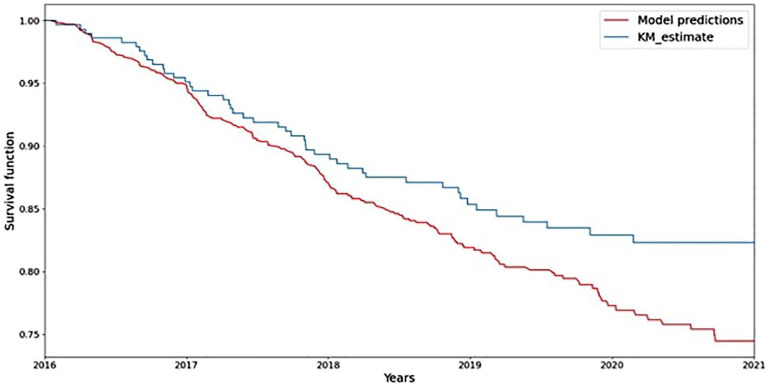
**Comparison of model predicted survival probabilities and observed survival rates using the Kaplan–Meier estimator**. The Kaplan–Meier estimator was employed as a benchmark using data from the test set.

For the estimation of the predictive performance of the model, the C-Index of the algorithm was 0.92 and IBS was 0.18 on the test set. When we calculated the Kaplan–Meier estimator using the same data from the test set and employed it as a benchmark, there was close agreement between the model’s predicted survival probabilities and observed survival rates in the test data (Figure1).

### Distribution and prediction of disability classes

**Figure 2.**
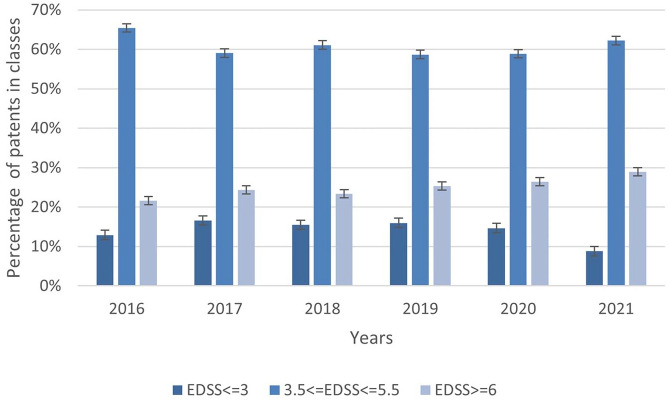
Distributions of predicted classes for each year from 2016 to 2021.

Figure 2 shows disability class distribution in Campania from 2016 to 2021. The progression rates between patients from the reference centre (*Multiple Sclerosis Clinical Care and Research Centre* of the University ‘Federico II’ of Naples (Italy)) and those from other centres were comparable (Supplemental Appendix 1). Overall, from 2016 to 2021, 9% of the population had EDSS ⩽ 3.0, 62% EDSS 3.5–5.5, and 29% EDSS ⩾ 6.0.

**Figure 3.**
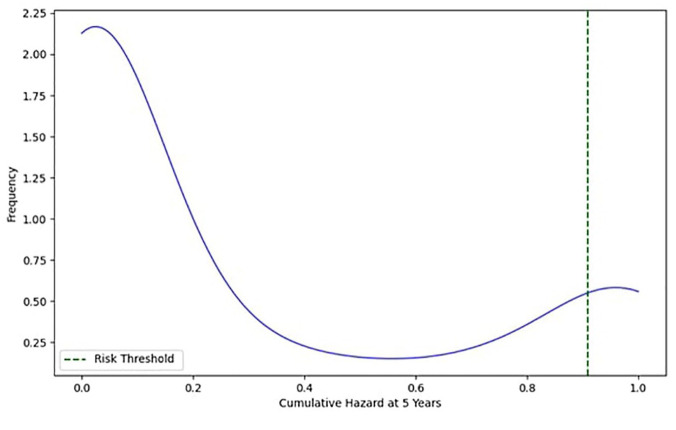
Distribution of cumulative risk with the high-risk threshold.

**Figure 4.**
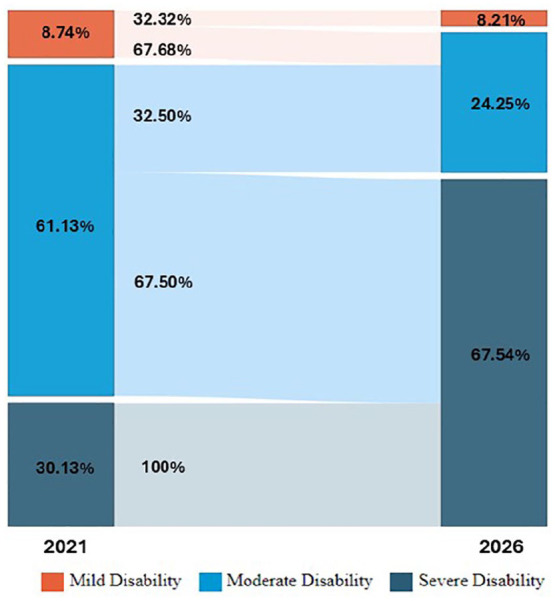
Projected disability progression among patients from 2021 to 2026.

Using k-means clustering, we estimated a risk threshold of 0.91 to identify patients at high risk of changing classes and thus progressing in disability. Figure 3 shows the distribution of the cumulative hazard over 5 years relative to the frequency of patients. The blue curve represents the distribution of the cumulative risk of patient progression. The graph suggests that patients with a cumulative hazard value above 0.91 are considered at high risk. Therefore, their progression in disability from 2022 to 2026 was visualized using a Sankey diagram, as shown in Figure 4, to better represent the transitions between different risk classes.

In 2021, 8.73% of patients had a lower level of disability, but, by 2026, only 2.82% are expected to remain in this category. The middle disability group comprised 61.1% of patients in 2021, with 25.8% anticipated to remain in this range by 2026. In contrast, 30.1% of patients in 2021 had more severe disability, and this percentage is projected to increase significantly to 71.4% by 2026, indicating notable disease progression over time.

## Discussion

We estimated MS-related disability and predicted its progression at the population level in a large Italian region with about 8000 PwMS, using a neural network algorithm based on routinely collected healthcare data validated towards a clinical registry. Furthermore, we estimated distribution of disability classes from 2016 to 2021 and projected progression risk from 2021 to 2026, providing a framework for real-time monitoring, healthcare planning, and intervention evaluation.

The neural network estimated EDSS with nearly 70% accuracy. Sensitivity and specificity across EDSS classes indicated that the model could effectively identify patients at different disability levels, with best performance in higher disability classes (EDSS 3.5–5.5 and ⩾6.0). These groups are within conventional milestones of disability progression, making their identification valuable for policy evaluation and/ treatment allocations. The hybrid model, combining survival analysis and neural networks, had a C-index of 0.92, confirming its predictive accuracy. Unlike traditional clinical registries, our algorithms equally weighted DMT use and other healthcare resources (e.g. hospital admissions, outpatient visits), targeting treated and untreated PwMS and including those with advanced disease stages. The accuracy of this algorithm could support improved treatment planning and healthcare delivery, to ultimately reduce disability progression.

Previous models estimating EDSS used clinical data or the self-reported sale, but depended on clinical data limiting their applicability.^6,22^ Some previous studies estimated EDSS using real-world data. A German study used ICD-10_GM code to estimate EDSS, achieving an F1-score of 0.68, identical to ours.^7^ Their study reported the highest sensitivity for low severity cases and lowest for moderate disability, while our algorithm performed best for moderate disability and less effectively for low disability. The differences in sensitivity between the two models may be due to the nature of the data used. Our algorithm showed higher accuracy for moderate disability, likely due to more frequent and heterogenous interactions with national healthcare services. We prioritized this class considering that one of the main objectives of the study was to derive an algorithm that could be used to improve treatment planning and allocation to delay disability progression.

The estimated disability distribution in our population aligns with global trends, as highlighted by recent Global Burden of Disease studies. These analyses indicate a steady increase in disability associated with neurological conditions over the years, coinciding with rising life expectancy worldwide.^23^ Furthermore, our model includes predictors from different domains, beyond the sole use of DMTs, strengthening its transferability in different settings, characterized by heterogeneous levels of healthcare resource utilization and where access to DMTs might vary.

These predictions could have significant implications for both clinical practice and future research, improving healthcare planning and medication supply chain.

This study distinguishes itself from others by employing high granularity datasets to estimate disability levels in PwMS. Moreover, the soundness of the adopted methodology is also ensured by employing a clinical registry for validation. The progression rate of patients from the reference centre is similar to that of patients from other centres. This demonstrates the robustness and generalizability of the algorithm, ensuring that the training method did not introduce bias in the algorithm’s predictions across different populations. Furthermore, this approach addresses the limitations of some previous efforts, which often lacked actual EDSS measures for validation.^24,25^ The algorithm uses a coding system which is based on the international classification of the disease system, and includes variables related to both MS centres (e.g. DMT prescription) and healthcare systems (e.g. emergency admissions). Therefore, the algorithm could potentially be adapted to different settings and conditions, offering the possibility for an external validation to evaluate its generalizability and reliability across diverse populations and healthcare systems. However, several caveats merit discussion.

A limitation of the study is the implementation of the analysis in a small geographical area. However, the Campania Region counts about 6 million inhabitants and is the third most populated region in Italy and the largest in Southern Italy. Furthermore, predicted prevalence of MS across Italian regions is fairly consistent.^23^ Therefore, findings might be translated to other Italian regions as well. A notable limitation of our study is the exclusion of 564 patients due to missing demographic data, representing more than 5% of the initial sample. This exclusion raises the potential risk of selection bias. However, a comparative analysis of key characteristics between the excluded sub-cohort and the main cohort indicated no significant differences.

In addition, our projection from 2021 to 2026 should be interpreted with caution, as it may not fully capture the evolving therapeutics and related impact on disability progression. The increasing use of high-efficacy DMTs in recent years may be gradually changing the natural course of the disease, potentially leading to a modest reduction in progression rates compared to earlier periods.

Another limitation of the study is that the algorithms were based on data collected during the study period and only included DMTs available in this window period. Hence, one DMT (Ofatumumab) that is available in Italy since 2022 has not been considered. However, the influence of DMTs on our model’s predictive power remains limited as our approach relies on clinical and administrative data rather than individual therapy effects.

A potential limitation of our study is the possibility of misdiagnosis, as some patients labelled as having MS in administrative databases may not meet the full clinical criteria. However, this issue represents a common limitation across studies relying on real-world or administrative data sources.

Finally, we focused on the EDSS to estimate disability at clinical level, which remains a cornerstone in clinical trials and observational studies when it comes to disability in MS. However, we acknowledge that additional measures could have been considered (e.g. upper limb function, cognitive impairment, and patient-reported outcome measures).

In conclusion, this study validated neural networks for MS disability estimation and progression risk, highlighting deep learning’s potential in disease-monitoring and healthcare planning.

## Supplemental Material

sj-docx-1-msj-10.1177_13524585251347513 – Supplemental material for Neural networks to estimate multiple sclerosis disability and predict progression using routinely collected healthcare data Supplemental material, sj-docx-1-msj-10.1177_13524585251347513 for Neural networks to estimate multiple sclerosis disability and predict progression using routinely collected healthcare data by Giuseppina Affinito, Marcello Moccia, Roberta Lanzillo, Ruth Ann Marrie, Jeremy Chataway, Vincenzo Brescia Morra and Raffaele Palladino in Multiple Sclerosis Journal
