# Supplementary material for: Neural networks to estimate multiple sclerosis disability and predict progression using routinely collected healthcare data
Source: Mult Scler. 2025 Jul 3;31(12):1417–26. doi: 10.1177/13524585251347513 (PMC12547042; doi:10.1177/13524585251347513)
Supplement: sj-docx-1-msj-10.1177_13524585251347513 – Supplemental material for Neural networks to estimate multiple sclerosis disability and predict progression using routinely collected healthcare data [file sj-docx-1-msj-10.1177_13524585251347513.docx]

**APPENDIX 1**

**Classification of Outpatient Services**

**Table 1** provides a comprehensive list of outpatient services categorized into Consultation, Diagnostic, Laboratory, Procedure, Therapeutic, and Rehabilitation. This classification was meticulously conducted by a group of expert neurologists to ensure accuracy and relevancy.

Table 1. List of outpatient services categorized into Consultation, Diagnostic, Laboratory, Procedure, Therapeutic, and Rehabilitation. This classification was meticulously conducted by a group of expert neurologists to ensure accuracy and relevancy.

| **Type** | **Description** |
| --- | --- |
| Consultation | Audiology consult |
| Consultation | Clinical nutrition consult |
| Consultation | Clinical nutrition follow-up consult |
| Consultation | Clinical psychological interview by session |
| Consultation | Consult for individual rehabilitation plan |
| Consultation | Consult for prosthetic plan |
| Consultation | Consult for recovery and functional re-education |
| Consultation | Dermatology assessment with epidiascope |
| Consultation | Dermatology assessment with epiluminscent microscopy |
| Consultation | Dermatology consult |
| Consultation | Dermatology follow-up consult |
| Consultation | Dermatology oncology consult |
| Consultation | Evaluation of the vibratory sensitivity threshold |
| Consultation | Eye consult / eye examination |
| Consultation | Eye follow-up consult |
| Consultation | Facial nerve paralysis |
| Consultation | Global functional assessment |
| Consultation | Infection consult |
| Consultation | Infection follow-up consult |
| Consultation | Mononeuritis of the limb, traumatic and non-traumatic, and multiple neuritis |
| Consultation | Multidisciplinary evaluation for multiple sclerosis |
| Consultation | Multidisciplinary evaluation for the treatment |
| Consultation | Neuropsychological tests for high cortical function |
| Consultation | Neuropsychological tests for pre-treatment evaluation of language disorders |
| Consultation | Neurology consult |
| Consultation | Neurology follow-up consult |
| Consultation | Nutritional support consult |
| Consultation | Nutritional support follow-up consult |
| Consultation | Pain follow-up consult |
| Consultation | PM&R consult |
| Consultation | PM&R follow-up consult |
| Consultation | Prosthetic evaluation |
| Consultation | Psychiatry consult |
| Consultation | Psychiatry follow-up consult |
| Consultation | Psychiatric interview |
| Consultation | Soft tissue diseases related to overuse |
| Consultation | Specific joint and muscular balance (hip, thigh, knee, etc) |
| Consultation | Urology consult |
| Consultation | Urinary and fecal incontinence |
| Diagnostic | 24h dynamic EEG |
| Diagnostic | Anorectal manometry (anorectal biofeedback) |
| Diagnostic | Aphasia examination |
| Diagnostic | Auditory evoked potential |
| Diagnostic | Auditory evoked potentials |
| Diagnostic | Auditory evoked potentials: threshold |
| Diagnostic | Computerized visual field (COWA) |
| Diagnostic | Computerized visual field (Humphrey) |
| Diagnostic | Computerized visual field (Octopus) |
| Diagnostic | Cystoscopy [transurethral] |
| Diagnostic | Cystoscopy [transurethral] with biopsy |
| Diagnostic | Cystometrography / cystomanometry |
| Diagnostic | EEG |
| Diagnostic | EEG with sleep deprivation |
| Diagnostic | EEG with video recording |
| Diagnostic | Fundus photographs |
| Diagnostic | General joint and muscular balance |
| Diagnostic | Hess - Lancaster test |
| Diagnostic | Manual visual field |
| Diagnostic | Motor evoked potentials: left arm |
| Diagnostic | Motor evoked potentials: left leg |
| Diagnostic | Motor evoked potentials: right arm |
| Diagnostic | Motor evoked potentials: right leg |
| Diagnostic | Motor evoked potentials: upper or lower limb |
| Diagnostic | MRI brain |
| Diagnostic | MRI brain (with and without contrast agent) |
| Diagnostic | MRI brain and brainstem |
| Diagnostic | MRI brain and brainstem (with and without contrast agent) |
| Diagnostic | MRI brain with neuronavigation |
| Diagnostic | MRI brain with neuronavigation system (with and without contrast agent) |
| Diagnostic | MRI brain: stereotactic system |
| Diagnostic | MRI brain: stereotactic system (with and without contrast agent) |
| Diagnostic | MRI cervical spine (with and without contrast agent) |
| Diagnostic | MRI dorsal spine (with and without contrast agent) |
| Diagnostic | MRI face |
| Diagnostic | MRI face (with and without contrast agent) |
| Diagnostic | MRI lumbosacral spine (with and without contrast agent) |
| Diagnostic | MRI whole spine |
| Diagnostic | MRI whole spine (with and without contrast agent) |
| Diagnostic | Neurophysiological tests for autonomic system |
| Diagnostic | Neuropsychological executive testing |
| Diagnostic | Neuropsychological intelligence test |
| Diagnostic | Neuropsychological memory testing |
| Diagnostic | Neuropsychological projective and personality tests |
| Diagnostic | Neuropsychological tests for cognitive impairment or development |
| Diagnostic | Neuropsychological visuospatial testing |
| Diagnostic | Partial eye examination (lens prescription) |
| Diagnostic | Posturographic test |
| Diagnostic | Reflex response |
| Diagnostic | Somato-sensory evoked potentials |
| Diagnostic | Static and dynamic stabilometric test |
| Diagnostic | Study of adaptability to the dark |
| Diagnostic | Study of color sensitivity |
| Diagnostic | Study of eye motility |
| Diagnostic | Study of provocated registered nystagmus |
| Diagnostic | Study of sensitivity to contrast agent |
| Diagnostic | Study of spontaneous or positional registered nystagmus |
| Diagnostic | Study of the visual field |
| Diagnostic | Study of the visual field: campimetry, static/kinetic perimetry, FDT |
| Diagnostic | Tensilon test |
| Diagnostic | Visual evoked potentials (pattern) |
| Diagnostic | Wechsler memory scale test |
| Laboratory | ALT (SGPT) |
| Laboratory | Alpha-fetoprotein (AFP) |
| Laboratory | Antibody cytomegalovirus (titration by FC) |
| Laboratory | Capillary blood sample |
| Laboratory | Cell blood count |
| Laboratory | Creatinine |
| Laboratory | Creatinine [24h urine] |
| Laboratory | Creatinine [amniotic liquid] |
| Laboratory | Creatinine [serum] |
| Laboratory | Creatinine [urine] |
| Laboratory | Creatinine clearance |
| Laboratory | Culture mycobacteria: biochemical tests |
| Laboratory | Culture mycobacteria: hybridization |
| Laboratory | EBV antibodies (EA or EBNA or VCA) (EIA) |
| Laboratory | EBV antibodies (EA or EBNA or VCA) (titration by IF) |
| Laboratory | EBV antibodies [R. Paul Bunnell Davidson] |
| Laboratory | EBV heterophilic antibodies (rapid test) |
| Laboratory | Free thyroxine (FT4) |
| Laboratory | Haematocrit |
| Laboratory | HBV-DNA hybridization (direct hybridization) |
| Laboratory | HBV-DNA hybridization (PCR) |
| Laboratory | HBV-DNA-polymerase |
| Laboratory | HBV-HBcAg antibody |
| Laboratory | HBV-HBcAg IgM antibody |
| Laboratory | HBV-HBeAg antibody |
| Laboratory | HBV-HBsAg antibody |
| Laboratory | HBV-HBsAg antibody (confirmatory test) |
| Laboratory | HCV antibody |
| Laboratory | HCV genomic typing |
| Laboratory | HCV immunoblotting (confirmatory test) |
| Laboratory | HCV quantitative test |
| Laboratory | HCV RNA qualitative analysis |
| Laboratory | Hepatitis delta virus (HDV): antibodies |
| Laboratory | Hepatitis delta virus (HDV): HDVAg antigen |
| Laboratory | Hepatitis delta virus (HDV): IgM antibodies |
| Laboratory | Herpes simplex virus (type 1 or 2): antibodies (IgG) |
| Laboratory | Herpes simplex virus (type 1 or 2): antibodies (IgM) |
| Laboratory | HIV 1 immunoblotting antibodies (confirmatory test) |
| Laboratory | HIV 1 p24 antigen |
| Laboratory | HIV 1 p24 antigen lymphocytes |
| Laboratory | HIV 1-2 antibodies |
| Laboratory | HIV 1-2 antibodies immunoblotting (confirmatory test) |
| Laboratory | HIV p24 antigen |
| Laboratory | HIV-1 RNA qualitative analysis |
| Laboratory | IGA, IGG, or IGM immunoglobulin |
| Laboratory | IgG subclasses: IgG1, IgG2, IgG3, and IgG4 |
| Laboratory | Interferon |
| Laboratory | Intestinal parasites: culture test |
| Laboratory | Intestinal parasites: macro and microscopic search |
| Laboratory | Intestinal parasites: microscopic search |
| Laboratory | Leucocytes (count) |
| Laboratory | Leucocytes (count and formula) |
| Laboratory | Microalbuminuria |
| Laboratory | Microbiological sample |

**Severe disability conditions**

**Table 2** presents specific ICD-9 codes used to identify severe disability conditions as referenced in the study.

Table 2. Specific ICD-9 codes identifying severe disability conditions.

| ICD-9 | Descr_icd_9 | Severity |
| --- | --- | --- |
| 2941 | Dementia in conditions classified elsewhere without behavioral disturbance | Severe |
| 3449 | Paralysis, unspecified | Severe |
| 3690 | Profound impairment, both eyes, impairment level not further specified | Severe |
| 3691 | Better eye: total vision impairment; lesser eye: total vision impairment | Severe |
| 7876 | Incontinence of feces | Severe |
| 7812 | Abnormality of gait | Severe |
| 7197 | Difficulty in walking | Severe |

**Sensitivities and Specificities with 95% Confidence Intervals (CI) for Different EDSS Classes**

Table 3. **Sensitivities and Specificities with 95% Confidence Intervals (CI) for Different EDSS Classes.**

|  | **Sensitivities (95% CI)** | **Specificities (95% CI)** |
| --- | --- | --- |
| **EDSS<=3** | 0.30 (0.24; 0.34) | 0.95 (0.92, 0.97) |
| **3.5<=EDSS<=5.5** | 0.91 (0.88, 0.94) | 0.50 (0.44, 0.56) |
| **EDSS>=6** | 0.48 (0.43, 0.54) | 0.92 (0.88, 0.95) |

Table 2. Comparison of Key Characteristics Between Excluded Sub-cohort and Main Cohort

|  | **Main Cohort (n=6,295)** | **Excluded Sub-cohort (n=564)** |  |
| --- | --- | --- | --- |
| **DMTs**  **(#ITPs/#patients)** |  |  |  |
| MABs | 1141 (18%) | 105 (19%) | $\chi^{2}=2.52$  $p=0.987$ |
| Oral | 3253 (51%) | 315 (56%) |  |
| Injectables | 2706 (42%) | 235 (42%) |  |
|  |  |  |  |
| Platform | 4744 (75%) | 423 (75%) | $\chi^{2}=0.01$  $p=0.562$ |
| High-Efficacy | 2355 (37%) | 215 (38%) |  |
|  |  |  |  |
| DMTs switches  (Platform to High-Efficacy) | 730 (11%) | 67 (12%) | $\chi^{2}=5.61$  $p=0.987$ |
| **Regular hospital admissions**  **(#admissions/#patients)** |  |  |  |
| Emergency admissions | 1,235(19%) | 115 (20%) | $\chi^{2}=1.32$  $p=0.147$ |
| MS-related admissions | 1,204(19%) | 135 (24%) | $\chi^{2}=20.6$  $p=0.962$ |
| **Outpatient services**  **(#outpatients/#patients)** |  |  |  |
| Consultation | 11,214(178%) | 1005 (178%) | $\chi^{2}=0.02$  $p=0.981$ |
| Diagnostic | 13,028(206%) | 1245 (221%) |  |
| Laboratory | 106,874(1697%) | 9685 (1717%) |  |
| Procedure | 1,137(18%) | 156 (28%) |  |
| Therapeutic | 39(1%) | 6 (1%) |  |
| **Rehabilitation outpatient services** | 2,455(38%) | 225 (40%) | $\chi^{2}=3.56$  $p=0.865$ |

**Table A1: Comparison of progression rates between the reference clinical centre (Multiple Sclerosis Clinical Care and Research Centre of the University “Federico II” of Naples (Italy)) and other centres**

|  | **Other Centres** | **Reference Centre** |
| --- | --- | --- |
| **No Progression (%)** | 1,229 (32.89%) | 404 (31.29%) |
| **Progression (%)** | 2,508 (67.11%) | 887 (68.71%) |
